# Supplementary material for: Comprehensive and clinically accurate head and neck cancer organs-at-risk delineation on a multi-institutional study
Source: Nat Commun. 2022 Oct 17;13:6137. doi: 10.1038/s41467-022-33178-z (PMC9576793; doi:10.1038/s41467-022-33178-z)
Supplement: Supplementary file 1 — Supplementary Information [file 41467_2022_33178_MOESM1_ESM.pdf]

Supplementary materials

Supplementary Method

Detailed segmentation baseline and auto-searched network architecture

The UNet backbone adopted in our work, i.e., the residual connection implementation of nnUNet [1], includes a 5-block encoding path and a 4-block decoding path. Each encoding block consists of the following consecutive operations with the residual connection: a convolution, a instance normalization [2], a Leaky ReLu unit, followed by a 2x2x2 max-pooling operator. Each decoding block is composed of a transposed convolution layer for up-sampling, followed by consecutive operations similar to the ones in the encoding block. The specific convolution operation in each block is automatically determined using the method of network architecture search (NAS) [3,4] with the search space defined by 2D, 3D, pseudo-3D (P3D) convolutions with kernel size of 3 or 5. The detailed convolutional neural network architectures for each organ at risk (OAR) segmentation branch are described in Supplementary Fig 1.

Implementation details

Image preprocessing. A windowing of [-500, 1000] HU to every pCT scan is applied covering the intensity range of our target OARs. VOIs of 256×256×64 voxels are randomly extracted around the OAR foreground as training samples for NAS. The heat map labels in the detection module are 3D Gaussian distributions (zero mean with standard deviation of 8mm) centered at the center of each S&H OAR.

Data split for NAS training and ablation study. We divide the training-validation dataset (176 patients from CGMH) into two subgroups for the NAS training and the ablation evaluation: 80% to train and validate the segmentation model and 20% as a held-out test set to evaluate the ablation performance. To avoid biases in selection of the learnable logits α_k when training NAS, we use a larger proportion of patients as validation than is typical, i.e., a validation/training

ratio of 1:2 for NAS. Therefore, when considering all 176 patients, the NAS training procedure uses 53% (80%×2/3) for training, 27% (80%×1/3) for validation, and 20% for ablation-testing (never seen in the NAS training). After finalizing the network architecture by the NAS procedure, we retrain the model from scratch using only the searched architecture and set the validation/training sizes to a more typical ratio: 64% for training, 16% for validation, and 20% for ablation-testing. More importantly, please note that the ablation-testing cases (20% of the Training-Validation dataset) were never seen in the NAS training and validation process.

NAS training. We exploit NAS to search the optimal network architecture for each stratified OAR segmentation branch. The combined Dice and Cross-Entropy losses are adopted, and the stochastic gradient descent optimizer is used with a Nesterov momentum of 0.99. To train the NAS parameter α_k, we first fix α_k to 1/9 for 400 epochs. Then we alternatively update α_k and the network weights for another additional 600 epochs. The batch size is set to 2 for NAS training. Only the validation set is used for updating α. The ratio between the training set and the validation set is 2:1. The initial learning rates are set to 0.01 for the anchor and mid-level branches, and 0.005 for the S&H branch, respectively. The learning rate is decayed following the Polynomial learning rate policy.

Final segmentation network training. After NAS is completed, we retrain the searched segmentation network from scratch. Data augmentation is applied [1], e.g., horizontal flipping, random rotations in the x-y plane within ±10 degrees, intensity scaling with a ratio between [0.75, 1.25], adding Gaussian noise with zero mean and (0, 0.1) variance. The batch size is 2. The optimizer is stochastic gradient descent with a Polynomial learning rate policy. The initial learning rate is 0.01 with a Nesterov momentum of 0.99. The S&H detection branch is trained using L2 loss with a 0.01 learning rate. The total number of training epochs for each module is 1000. The average training time is 9~10 GPU days. For inference, the average running time is normally less than 3 minutes per patient. All deep models are developed using PyTorch and trained on one NVIDIA Quadro RTX 8000 GPU.

Quantitative ablation results of SOARS in the training-validation dataset

Effect of processing stratification in SOARS. Processing stratification played a key role to improve the OAR segmentation performance. The processing stratification ablation results are shown in Table 2. The baseline is using 3D UNet model (implemented in the nnUNet framework)[1] trained on all 42 OARs together. When anchor OARs were stratified to train only on themselves, there was a 2.4% Dice similarity coefficient (DSC) improvement as compared to the baseline models. When focusing on mid-level OARs, with the help of anchor OAR guidance, there was a significant 37% Hausdorff distance (HD) error reduction (11.4 versus 18.0mm) as compared to the baseline model of training on all OARs. This demonstrated the intrinsic difficulty in segmenting a large number of various organs without explicitly taking their differences into account. It simultaneously indicated that anchor OARs served as effective references to better delineate the hard-to-discern boundaries of mid-level organs (most are soft-tissue organs). For S&H OARs, by cropping the volume of interest (VOI) using the detection module and with the support of anchor OAR predictions, there were remarkable accuracy improvements in segmenting S&H OARs, boosting DSC from 58.3% to 73.7%, as compared against directly segmenting from CT. This further demonstrated the merits and advantages of our stratified learning approach that adapted to provide the optimal handling of OAR categories with different characteristics. Fig. 3 depicts qualitative examples of segmenting anchor, mid-level and S&H OARs.

Effect of neural architecture search (NAS) associated with SOARS. Table 2 also outlines the performance improvements provided by NAS. As can be seen, all three branches trained with NAS consistently produced more accurate segmentation results than those trained using the baseline 3D UNet. This validated the effectiveness of NAS on more complicated segmentation tasks. For the three branches, mid-level and S&H OAR categories showed considerable performance improvements, from 72.6% to 74.2% and 73.7% to 76.2% in DSC

scores respectively, while the anchor branch provides a marginal but consistent improvement (0.7% in DSC). Considering that anchor OARs are already relatively easy to segment, the fact that NAS can further boost the performance attested to its benefits.

The NAS searched neural network architectures are depicted in Supplementary Fig. 1. It is observed that, for the encoding path, the mid-level and S&H branches gradually involve more 3D or P3D convolution kernels as compared to the anchor branch. This indicates that 3D kernels may not always be the best choice for segmenting objects with reasonable size or contrast, as 2D kernels dominate the anchor branch. Consequently, appropriate 2D and P3D kernels can reduce the computation cost and memory consumption. For the S&H branch, our findings are consistent with the intuition that small or low contrast objects rely more on the 3D spatial information and context for better segmentation. As for the decoding path, all three branches are mainly equipped with 3D or P3D convolution kernels. This is an interesting result, as it implies that the decoding path tries to incorporate the convolutional features in a more 3D fashion for all three OAR categories.

Blinded user study to assess the OAR editing efforts

We have further designed another blind user study to assess the observer variation in evaluating the OAR editing efforts. In this blind user study, we used 30 multiuser testing patients from FAH-ZU and involved the senior physician (J. Ge) who has originally drawn the gold-standard contours of 13 OAR types in these patients. For each OAR, we randomly selected its contour from three OAR sources {gold-standard, SOARS, or the other human reader} (see supplementary Fig. 6) and presented it to this physician blindly. The true contour source for each OAR was kept unknown to the physician. We asked the physician to judge if each OAR contour needs editing or not. We report the number of OAR contours required for editing for each OAR source. Results are shown in the supplementary Table 9. From the blind user study, it is observed that there are 15% of the gold-standard contours were deemed requiring further

editing, which reflects the intra-observer variation on assessing the OAR revision efforts. For SOARS contours, 43% requires revision, which is slightly higher than that in the original unblind assessment by this physician where 37% SOARS contours required revision among the 13 OAR types of FAH-ZU. Since the required revision number of SOARS contours from the blind vs unblind assessment two times' study is close, it indicates that our observer variation/bias is within a small range. Moreover, compared with SOARS, a noticeably higher number of human reader's contours requires revision (55% vs 43% of SOARS), reflecting that SOARS contours' quality is generally better than the human reader's in the blind assessment. This observation is also consistent with that seen in the quantitative contouring accuracy between SOARS and the human reader (Table 5 in the main manuscript text). This additional analysis further strengthens our results regarding the OAR editing efforts.

Supplementary Figures

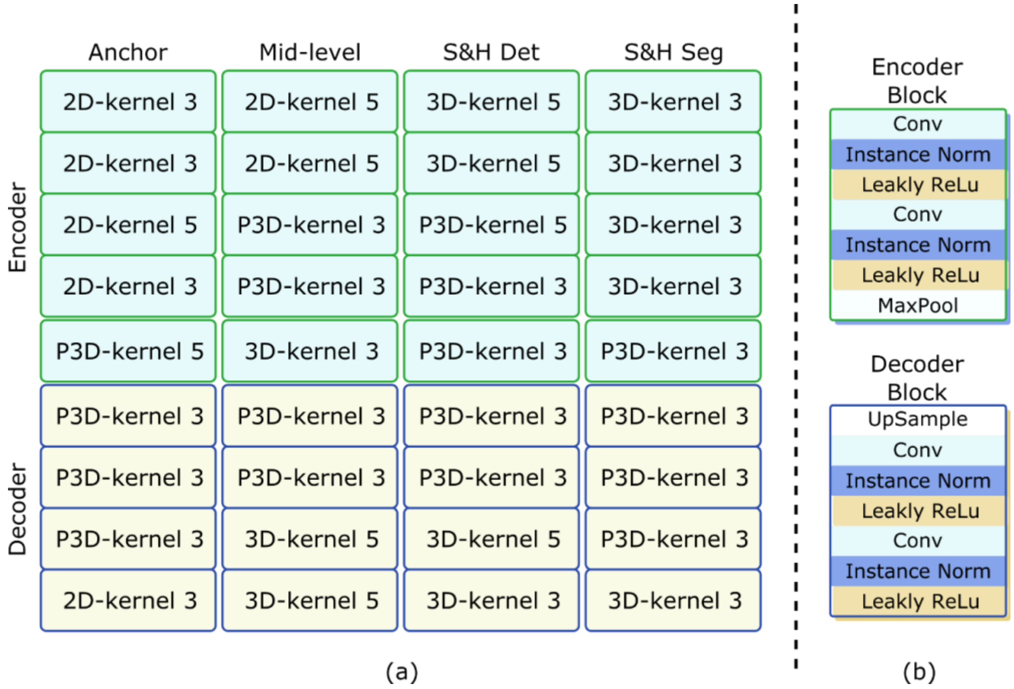

Supplementary Fig. 1 The detailed auto-searched backbone network architecture based on UNet. (a) illustrates the auto-searched network architecture for the anchor, mid-level, and small & hard (S&H) branches. The search space of the convolution operation includes 2D, 3D, and pseudo-3D (P3D) with either kernel size of 3 or 5. (b) lists the detailed operations in the encoder and decoder blocks. The auto-searched two convolution operations within each block are of the same type.

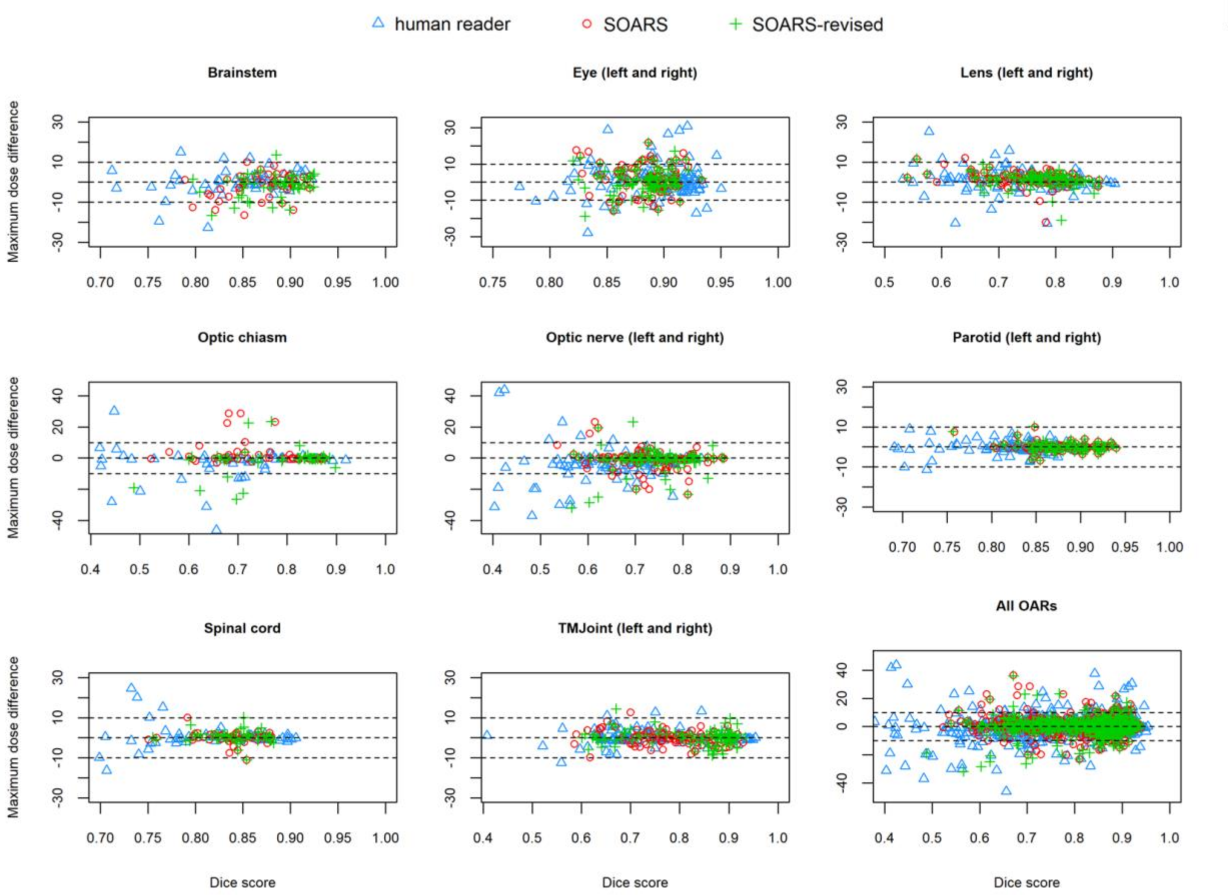

Supplementary Fig. 2. The scatter plots of direct maximum dose differences ($\mathrm{Diff}_{\mathrm{max\,dose}}^{\mathrm{direct}}$ in Equation 7) brought by various OAR contour sets of SOARS, SOARS-revised, and human reader when using the original IMRT dose grids in 50 multi-user testing patients. Each OAR and all OAR results are plotted, respectively. Blue triangle, green cross and red circle represent the results of human reader, SOARS-revised and SOARS, respectively.

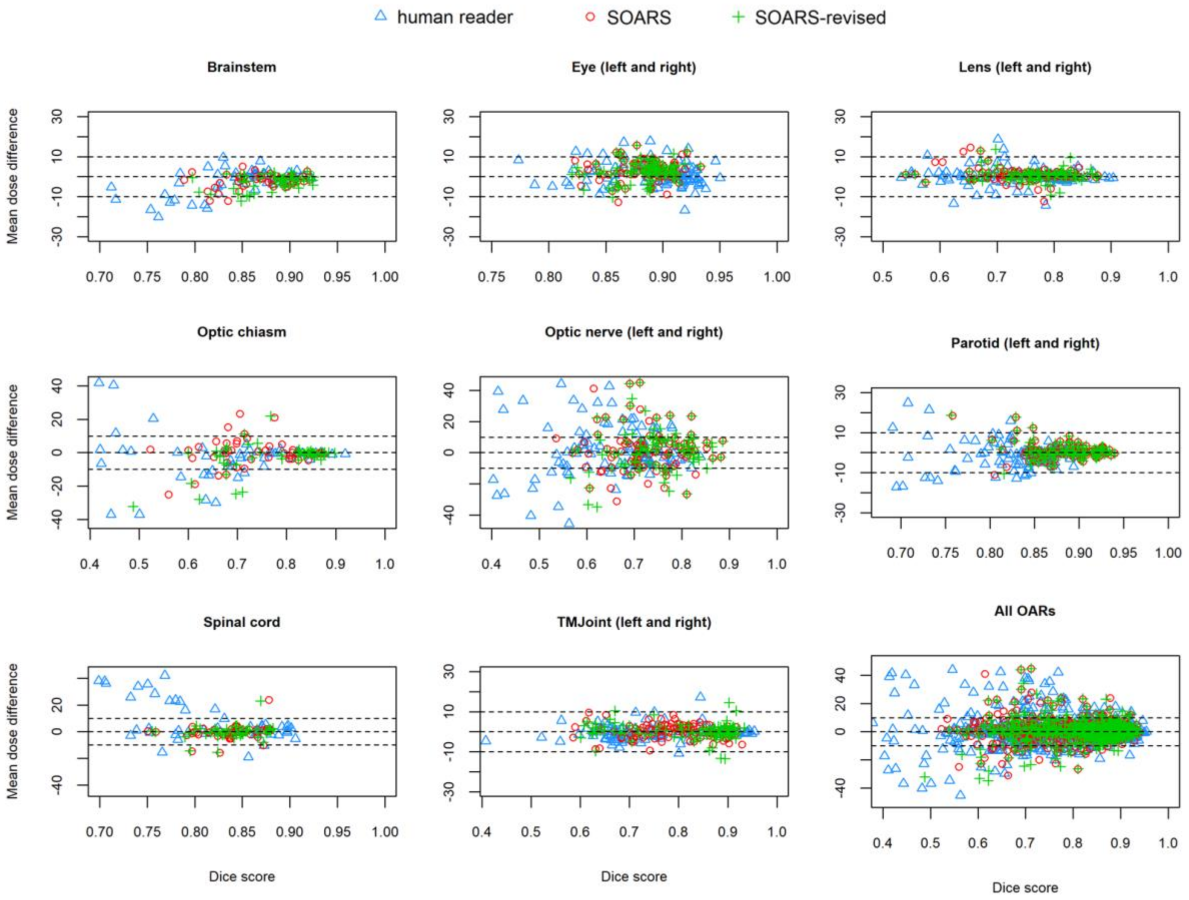

Supplementary Fig. 3. The scatter plots of direct mean dose differences ($\mathrm{Diff}_{\mathrm{mean\ dose}}^{\mathrm{direct}}$ in Equation 6) brought by various OAR contour sets of SOARS, SOARS-revised, and human reader when using the original IMRT dose grids in 50 multi-user testing patients. Each OAR and all OAR results are plotted, respectively. Blue triangle, green cross and red circle represent the results of human reader, SOARS-revised and SOARS, respectively.

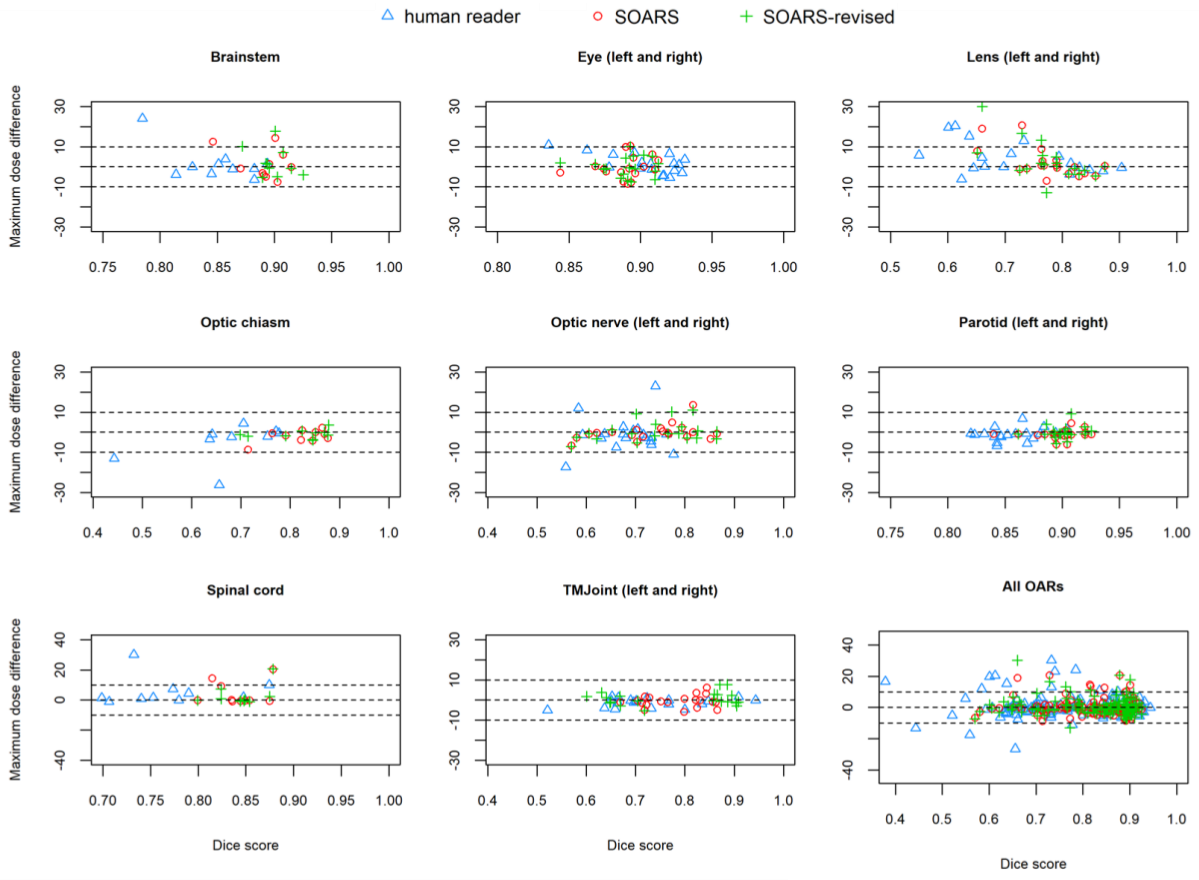

Supplementary Fig. 4. The scatter plots of clinical maximum dose differences ($\mathrm{Diff}_{\mathrm{max\,dose}}^{\mathrm{clinical}}$ in Equation 9) in 10 randomly selected multi-user testing patients from FAH-ZU, where the new IMRT planning dose grids were generated by using the original tumor target volumes and the substitute OAR contours (SOAR, SOARS-revised, and human reader), and then, the clinical reference OAR contours were overlaid on top of each new dose grid. Each OAR and all OAR results are plotted, respectively. Blue triangle, green cross and red circle represent the results of human reader, SOARS-revised and SOARS, respectively.

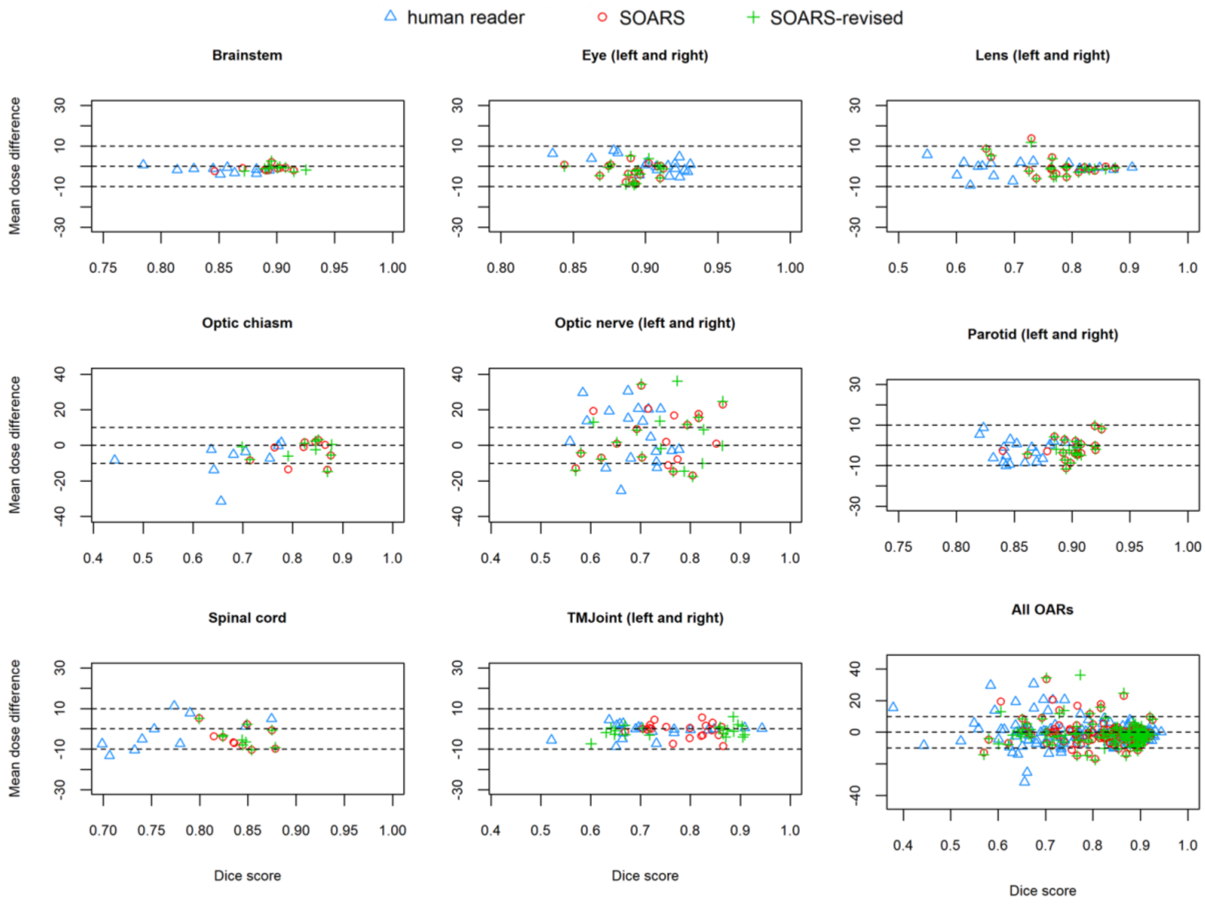

Supplementary Fig. 5. The scatter plots of clinical mean dose differences ($\mathrm{Diff}_{\text{mean dose}}^{\text{clinical}}$ in Equation 8) in 10 randomly selected multi-user testing patients from FAH-ZU, where the new IMRT planning dose grids were generated by using the original tumor target volumes and the substitute OAR contours (SOAR, SOARS-revised, and human reader), and then, the clinical reference OAR contours were overlaid on top of each new dose grid. Each OAR and all OAR results are plotted, respectively. Blue triangle, green cross and red circle represent the results of human reader, SOARS-revised and SOARS, respectively.

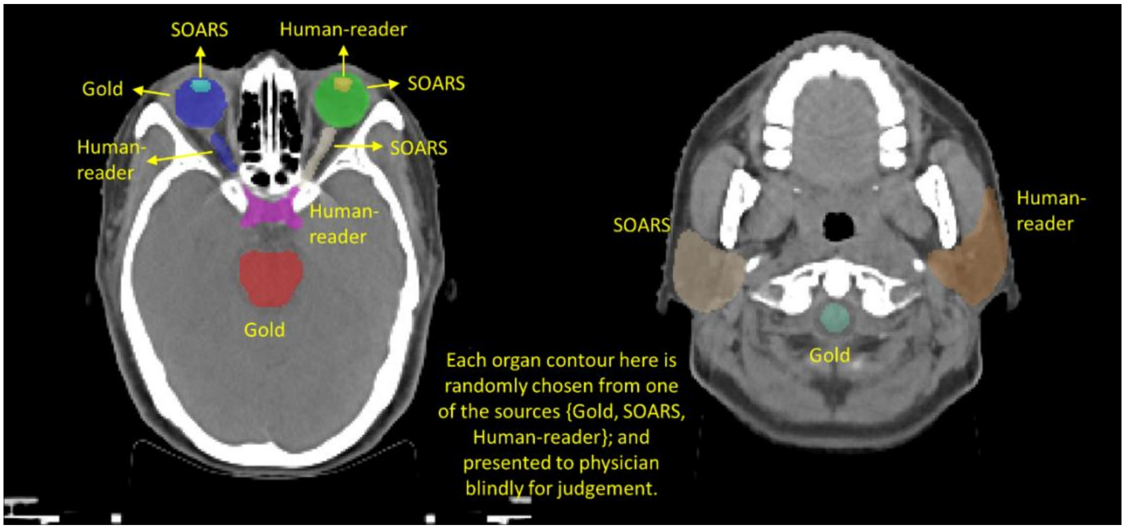

Supplementary Fig. 6. Examples of randomly selected OARs in the blind user study for the observer variation/bias assessment in evaluating the OAR editing efforts. Each OAR in a patient is randomly chosen from one of the three contouring sources {Gold, SOARS, Human-reader}. These OAR contours are presented blindly to the physician to determine if revision for any of the OARs are needed.

Supplementary Tables

Supplementary Table 1. Detailed planning CT imaging protocols in each institution. CE represents contrast-enhanced; NC represents non-contrast.

	CGMH (n = 502)	FAH-XJU (n = 82)	FAH-ZU (n = 447)	GPH (n = 50)	HHA-FU (n = 195)	SMU (n = 227)
Scanner make	GE	Philips	Siemens	GE	Siemens	Philips
NC or CE	NC CE mixed	NC CE mixed	NC	CE	NC CE mixed	NC
Scanning parameter (voltage and current)	120kV 300mAs	120kV 280mAs	120kV 300mAs	120kV 280mAs	120kV 280mAs	120kV 275-375mAs
Spatial resolution (mm)						
Median	0.99×0.99×2.5	0.94×0.94×3.0	0.98×0.98×3.0	0.8×0.8×3.0	0.97×0.97×3.0	0.53×0.53×3.0
Minimum	0.84×0.84×1.0	0.8×0.8×1.0	0.82×0.82×1.5	0.7×0.7×3.0	0.41×0.41×1.0	0.44×0.44×3.0
Maximum	1.37×1.37×3.0	1.19×1.19×3.0	1.27×1.27×3.0	0.98×0.98×3.0	0.98×0.98×5.0	0.64×0.64×3.0

Supplementary Table 2. Quantitative comparisons on the external FAH-XJU testing dataset of 82 patients. The proposed SOARS outperforms the previous leading approach UaNet in nearly all metrics across different OARs. DSC, HD and ASD represent Dice similarity coefficient, Hausdorff distance, and average surface distance, respectively.

OARs	UaNet			SOARS		
	DSC	HD (mm)	ASD (mm)	DSC	HD (mm)	ASD (mm)
BrainStem	78.4% ± 6.4%	9.2 ± 3.1	1.9 ± 0.8	**80.5% ± 6.7%**	**8.2 ± 3.0**	**1.8 ± 0.9**
Eye_Lt	**86.7% ± 5.3%**	**5.0 ± 5.3**	**0.8 ± 0.6**	85.8% ± 5.7%	5.4 ± 5.4	0.9 ± 0.7
Eye_Rt	**87.5% ± 2.6%**	**4.1 ± 1.0**	**0.7 ± 0.2**	86.1% ± 3.5%	4.3 ± 1.2	**0.7 ± 0.3**
Lens_Lt	68.4% ± 9.4%	2.8 ± 0.6	0.6 ± 0.3	**69.6% ± 7.0%**	**2.7 ± 0.6**	**0.6 ± 0.2**
Lens_Rt	70.8% ± 8.2%	2.8 ± 0.7	0.5 ± 0.3	**72.9% ± 7.2%**	**2.4 ± 0.7**	**0.5 ± 0.2**
OpticChiasm	57.6% ± 14.0%	6.7 ± 2.4	1.5 ± 0.8	**68.1% ± 7.0%**	**6.5 ± 3.0**	**1.0 ± 0.5**
OpticNerve_Lt	66.0% ± 7.4%	**5.0 ± 2.6**	**0.8 ± 0.4**	**67.9% ± 6.9%**	6.2 ± 3.0	**0.8 ± 0.3**
OpticNerve_Rt	65.5% ± 8.5%	**4.3 ± 1.1**	**0.8 ± 0.3**	**66.4% ± 6.0%**	4.8 ± 1.2	**0.8 ± 0.2**
Parotid_Lt	78.2% ± 5.2%	11.7 ± 3.0	1.9 ± 0.6	**79.7% ± 5.0%**	**10.5 ± 3.2**	**1.8 ± 0.6**
Parotid_Rt	77.6% ± 6.2%	12.4 ± 4.5	2.0 ± 0.8	**79.4% ± 5.2%**	**10.9 ± 3.8**	**1.8 ± 0.6**
Pituitary	62.4% ± 12.8%	4.4 ± 1.5	1.2 ± 0.6	**75.6% ± 11.1%**	**3.7 ± 1.6**	**0.6 ± 0.5**
SpinalCord	79.2% ± 14.2%	7.5 ± 9.0	1.0 ± 1.2	**82.3% ± 4.9%**	**6.7 ± 9.4**	**0.9 ± 1.4**
TMJ_Lt	76.7% ± 5.2%	12.5 ± 6.5	1.9 ± 1.5	**77.8% ± 9.0%**	**10.9 ± 7.0**	**1.5 ± 1.5**
TMJ_Rt	72.8% ± 12.4%	11.3 ± 8.4	2.1 ± 1.6	**81.4% ± 5.2%**	**6.9 ± 1.5**	**0.9 ± 0.3**
**Average**	74.8%	7.2	1.2	**77.3%**	**6.4**	**1.0**

Note: Bold and highlighted values represent the best performance and statistically significant improvements calculated using Wilcoxon matched-pairs signed rank test as compared between UaNet and SOARS, respectively. Statistical significance is set at two-tailed $p<0.05$.

Supplementary Table 3. Quantitative comparisons on the external FAH-ZU testing dataset of 447 patients. The proposed SOARS outperforms the previous leading approach UaNet in nearly all metrics across different OARs. DSC, HD and ASD represent Dice similarity coefficient, Hausdorff distance, and average surface distance, respectively.

OARs	UaNet			SOARS		
	DSC	HD (mm)	ASD (mm)	DSC	HD (mm)	ASD (mm)
BrainStem	77.8% ± 10.9%	10.1 ± 6.2	2.6 ± 2.4	**82.4% ± 11.3%**	**8.3 ± 5.7**	**1.8 ± 2.2**
Eye_Lt	**87.9% ± 3.0%**	3.8 ± 0.9	0.6 ± 0.2	**87.9% ± 3.6%**	**3.5 ± 1.0**	**0.5 ± 0.3**
Eye_Rt	86.8% ± 5.6%	4.3 ± 5.7	0.9 ± 4.4	**87.3% ± 2.2%**	**3.7 ± 1.0**	**0.6 ± 0.2**
Lens_Lt	69.6% ± 10.5%	3.0 ± 1.1	0.7 ± 0.5	**71.4% ± 9.0%**	**3.0 ± 1.0**	**0.6 ± 0.4**
Lens_Rt	70.5% ± 10.7%	2.9 ± 1.2	0.7 ± 0.5	**72.0% ± 8.4%**	**2.9 ± 0.9**	**0.6 ± 0.4**
OpticChiasm	53.0% ± 15.7%	9.9 ± 5.8	2.3 ± 1.6	**65.9% ± 12.8%**	**6.6 ± 4.9**	**1.1 ± 0.6**
OpticNerve_Lt	**66.4% ± 9.7%**	8.9 ± 5.0	1.1 ± 1.9	66.3% ± 8.1%	**5.4 ± 2.9**	**0.7 ± 0.5**
OpticNerve_Rt	**68.3% ± 8.4%**	7.6 ± 3.9	0.8 ± 0.4	66.1% ± 7.8%	**5.3 ± 2.3**	**0.7 ± 0.3**
Parotid_Lt	82.2% ± 4.8%	12.8 ± 5.1	1.7 ± 0.7	**85.4% ± 4.6%**	**10.6 ± 4.7**	**1.2 ± 0.5**
Parotid_Rt	82.8% ± 5.2%	12.1 ± 6.0	1.6 ± 0.8	**84.8% ± 4.5%**	**11.1 ± 5.3**	**1.3 ± 0.7**
SpinalCord	83.8% ± 7.6%	13.1 ± 22.6	1.5 ± 6.0	**86.3% ± 7.4%**	**8.6 ± 22.0**	**1.3 ± 6.5**
TMJ_Lt	64.3% ± 8.5%	4.4 ± 1.0	1.3 ± 0.4	**76.2% ± 7.7%**	**3.7 ± 0.9**	**0.7 ± 0.4**
TMJ_Rt	63.5% ± 10.1%	4.5 ± 1.8	1.3 ± 0.5	**74.6% ± 7.8%**	**3.9 ± 1.7**	**0.8 ± 0.5**
**Average**	73.5%	7.5	1.3	**77.4%**	**5.9**	**0.9**

Note: Bold and highlighted values represent the best performance and statistically significant improvements calculated using Wilcoxon matched-pairs signed rank test as compared between UaNet and SOARS, respectively. Statistical significance is set at two-tailed p<0.05.

Supplementary Table 4. Quantitative comparisons on the external GPH testing dataset of 50 patients. The proposed SOARS outperforms the previous leading approach UaNet in nearly all metrics across different OARs. DSC, HD and ASD represent Dice similarity coefficient, Hausdorff distance, and average surface distance, respectively.

OARs	UaNet			SOARS		
	DSC	HD (mm)	ASD (mm)	DSC	HD (mm)	ASD (mm)
BrainStem	77.1% ± 14.6%	12.6 ± 7.2	2.4 ± 1.1	**78.9% ± 10.5%**	**11.7 ± 9.5**	**2.1 ± 1.4**
Eye_Lt	85.6% ± 3.5%	4.0 ± 0.8	0.8 ± 0.3	**92.1% ± 3.9%**	**3.5 ± 0.7**	**0.4 ± 0.3**
Eye_Rt	85.3% ± 4.6%	4.4 ± 1.2	0.8 ± 0.4	**91.5% ± 4.2%**	**3.3 ± 0.9**	**0.4 ± 0.3**
Lens_Lt	78.1% ± 8.2%	2.1 ± 0.7	0.4 ± 0.2	**82.2% ± 5.2%**	**2.1 ± 0.5**	**0.3 ± 0.2**
Lens_Rt	79.6% ± 9.4%	2.7 ± 0.9	**0.3 ± 0.2**	**81.9% ± 7.5%**	**2.0 ± 0.6**	**0.3 ± 0.2**
Mandible_Lt	89.8% ± 1.4%	**6.7 ± 2.7**	0.8 ± 0.2	**91.7% ± 1.1%**	**6.7 ± 2.7**	**0.7 ± 0.1**
Mandible_Rt	88.8% ± 1.2%	9.1 ± 2.1	0.8 ± 0.2	**91.8% ± 1.2%**	**6.2 ± 2.8**	**0.7 ± 0.1**
OpticChiasm	51.5% ± 16.0%	9.1 ± 2.1	2.2 ± 1.0	**60.1% ± 9.8%**	**7.7 ± 2.2**	**1.1 ± 0.5**
OpticNerve_Lt	57.9% ± 16.7%	6.4 ± 4.0	1.6 ± 1.4	**69.9% ± 6.1%**	**4.8 ± 1.5**	**0.6 ± 0.3**
OpticNerve_Rt	57.4% ± 18.5%	6.5 ± 3.5	1.6 ± 1.5	**69.2% ± 8.3%**	**4.6 ± 1.6**	**0.6 ± 0.3**
OralCavity	69.0% ± 3.1%	**23.6 ± 4.5**	5.3 ± 0.8	**72.2% ± 4.7%**	26.9 ± 4.9	**4.1 ± 0.8**
Parotid_Lt	87.1% ± 4.3%	11.3 ± 6.2	0.9 ± 0.5	**87.6% ± 4.4%**	**9.8 ± 5.8**	**0.8 ± 0.6**
Parotid_Rt	86.5% ± 4.5%	9.7 ± 5.8	0.9 ± 0.6	**87.1% ± 4.4%**	**8.8 ± 4.6**	**0.8 ± 0.5**
Pituitary	88.8% ± 3.3%	2.3 ± 0.8	**0.1 ± 0.1**	**89.0% ± 3.3%**	**2.0 ± 0.3**	**0.1 ± 0.1**
SpinalCord	78.7% ± 5.4%	6.6 ± 2.5	**1.1 ± 0.5**	**78.9% ± 5.1%**	**6.5 ± 2.5**	**1.1 ± 0.5**
TMJ_Lt	65.8% ± 17.1%	8.1 ± 4.6	1.5 ± 0.8	**73.1% ± 20.0%**	**4.1 ± 1.7**	**0.9 ± 0.9**
TMJ_Rt	65.0% ± 17.2%	7.2 ± 3.7	1.5 ± 0.8	**75.3% ± 23.2%**	**4.1 ± 1.7**	**0.8 ± 1.0**
**Average**	76.0%	7.6	1.4	**80.7%**	**6.8**	**0.9**

Note: Bold and highlighted values represent the best performance and statistically significant improvements calculated using Wilcoxon matched-pairs signed rank test as compared between UaNet and SOARS, respectively. Statistical significance is set at two-tailed $p<0.05$. When the mandible is considered as a single OAR instead of left and right mandible, SOARS achieves the mean DSC, HD and ASD of 91.8%, 6.4mm, and 0.7mm, respectively.

Supplementary Table 5. Quantitative comparisons on the external HHA-FU testing dataset of 195 patients. The proposed SOARS outperforms the previous leading approach UaNet in nearly all metrics across different OARs. DSC, HD and ASD represent Dice similarity coefficient, Hausdorff distance, and average surface distance, respectively.

OARs	UaNet			SOARS		
	DSC	HD (mm)	ASD (mm)	DSC	HD (mm)	ASD (mm)
BrainStem	75.8% ± 13.2%	13.3 ± 7.5	2.9 ± 1.8	**78.4% ± 8.9%**	**10.6 ± 6.8**	**2.3 ± 1.4**
Eye_Lt	85.3% ± 7.0%	4.0 ± 1.2	0.9 ± 0.5	**90.6% ± 5.7%**	**3.7 ± 1.1**	**0.5 ± 0.4**
Eye_Rt	86.3% ± 6.6%	3.8 ± 1.1	0.8 ± 0.5	**90.9% ± 5.6%**	**3.5 ± 1.1**	**0.5 ± 0.4**
Lens_Lt	78.4% ± 9.0%	2.3 ± 0.7	0.4 ± 0.3	**82.3% ± 6.9%**	**2.1 ± 0.6**	**0.3 ± 0.2**
Lens_Rt	78.2% ± 8.0%	2.3 ± 0.6	0.4 ± 0.2	**82.4% ± 6.7%**	**2.1 ± 0.6**	**0.3 ± 0.2**
OpticChiasm	50.1% ± 15.1%	10.7 ± 3.5	2.5 ± 1.2	**57.2% ± 10.2%**	**9.5 ± 2.9**	**1.5 ± 0.7**
OpticNerve_Lt	52.4% ± 14.7%	7.2 ± 4.1	1.6 ± 1.2	**62.3% ± 8.5%**	**6.2 ± 2.9**	**1.0 ± 0.4**
OpticNerve_Rt	56.2% ± 13.7%	**6.0 ± 3.1**	1.2 ± 0.8	**61.9% ± 9.7%**	6.2 ± 3.1	**1.0 ± 0.4**
Parotid_Lt	85.1% ± 6.0%	8.6 ± 4.1	1.1 ± 0.7	**85.6% ± 6.0%**	**7.7 ± 3.7**	**1.0 ± 0.7**
Parotid_Rt	84.1% ± 6.5%	10.1 ± 12.9	1.6 ± 4.2	**85.5% ± 5.8%**	**9.2 ± 12.9**	**1.3 ± 4.0**
SpinalCord	74.9% ± 12.6%	12.2 ± 26.7	1.9 ± 4.6	**78.9% ± 6.8%**	**7.6 ± 2.7**	**1.2 ± 0.6**
SMG_Lt	70.7% ± 5.2%	13.5 ± 2.1	2.2 ± 0.2	**78.3% ± 7.9%**	**7.9 ± 1.5**	**1.3 ± 0.3**
SMG_Rt	**76.2% ± 4.5%**	10.5 ± 6.4	1.6 ± 0.7	**76.2% ± 9.1%**	**7.5 ± 2.2**	**1.4 ± 0.9**
**Average**	73.2%	8.0	1.5	**77.7%**	**6.4**	**1.0**

Note: Bold and highlighted values represent the best performance and statistically significant improvements calculated using Wilcoxon matched-pairs signed rank test as compared between UaNet and SOARS, respectively. Statistical significance is set at two-tailed $p<0.05$.

Supplementary Table 6. Quantitative comparisons on the external SMU testing dataset of 227 patients. The proposed SOARS outperforms the previous leading approach UaNet in nearly all metrics across different OARs. DSC, HD and ASD represent Dice similarity coefficient, Hausdorff distance, and average surface distance, respectively.

OARs	UaNet			SOARS		
	DSC	HD (mm)	ASD (mm)	DSC	HD (mm)	ASD (mm)
BrainStem	78.7% ± 7.9%	12.6 ± 19.1	2.4 ± 3.2	**81.2% ± 7.2%**	**11.4 ± 19.9**	**2.1 ± 3.3**
Eye_Lt	85.8% ± 8.4%	3.8 ± 0.9	0.7 ± 0.3	**90.8% ± 4.7%**	**3.6 ± 0.9**	**0.5 ± 0.3**
Eye_Rt	86.6% ± 8.5%	3.7 ± 0.9	0.7 ± 0.3	**90.5% ± 4.7%**	**3.6 ± 0.9**	**0.5 ± 0.3**
InnerEar_Lt	55.1% ± 12.8%	8.0 ± 7.4	1.9 ± 1.0	**61.6% ± 14.0%**	**4.9 ± 2.0**	**0.9 ± 0.6**
InnerEar_Rt	54.0% ± 14.5%	9.4 ± 11.2	2.4 ± 2.4	**64.0% ± 13.8%**	**4.7 ± 1.9**	**0.8 ± 0.5**
Lens_Lt	81.1% ± 8.9%	2.1 ± 0.8	0.3 ± 0.2	**83.8% ± 5.9%**	**2.0 ± 0.7**	**0.2 ± 0.2**
Lens_Rt	80.1% ± 9.4%	**2.1 ± 0.8**	**0.3 ± 0.2**	**82.5% ± 7.6%**	**2.1 ± 0.8**	**0.3 ± 0.2**
Mandible_Lt	85.3% ± 12.5%	9.4 ± 9.0	1.5 ± 2.7	**88.8% ± 3.5%**	**7.7 ± 7.6**	**1.2 ± 1.0**
Mandible_Rt	85.7% ± 7.2%	9.5 ± 8.2	1.3 ± 1.2	**89.1% ± 3.3%**	**7.8 ± 7.8**	**1.2 ± 1.0**
OpticChiasm	53.0% ± 15.3%	6.6 ± 2.0	1.4 ± 0.7	**69.1% ± 10.9%**	**5.8 ± 2.1**	**0.6 ± 0.4**
OpticNerve_Lt	63.9% ± 13.9%	5.7 ± 5.4	1.0 ± 1.2	**69.0% ± 7.6%**	**4.8 ± 2.4**	**0.6 ± 0.4**
OpticNerve_Rt	64.7% ± 14.7%	5.5 ± 4.6	1.0 ± 1.4	**68.8% ± 8.1%**	**4.6 ± 1.8**	**0.6 ± 0.3**
OralCavity	48.2% ± 6.9%	29.4 ± 7.3	9.0 ± 1.7	**50.9% ± 6.5%**	**28.2 ± 5.0**	**7.5 ± 1.4**
Parotid_Lt	85.0% ± 6.7%	10.6 ± 10.3	1.0 ± 0.9	**87.4% ± 4.3%**	**9.6 ± 10.9**	**0.7 ± 0.5**
Parotid_Rt	83.3% ± 8.0%	12.4 ± 11.7	1.4 ± 2.7	**87.6% ± 4.5%**	**10.4 ± 11.1**	**0.8 ± 0.8**
Pituitary	66.7% ± 15.2%	4.2 ± 1.3	0.9 ± 0.7	**73.2% ± 10.1%**	**3.7 ± 1.0**	**0.5 ± 0.4**
SpinalCord	80.3% ± 11.4%	6.2 ± 5.4	0.8 ± 0.6	**83.0% ± 4.8%**	**4.6 ± 1.3**	**0.7 ± 0.2**
SMG_Lt	70.9% ± 2.1%	5.4 ± 0.8	2.6 ± 0.2	**75.3% ± 0.7%**	**4.9 ± 1.9**	**1.3 ± 0.1**
SMG_Rt	**74.2% ± 2.7%**	**5.9 ± 3.4**	1.3 ± 0.1	73.4% ± 1.7%	6.6 ± 0.7	**1.0 ± 0.0**
TempLobe_Lt	75.6% ± 4.1%	22.5 ± 6.8	2.6 ± 1.1	**78.8% ± 3.1%**	**20.6 ± 5.5**	**2.2 ± 0.9**
TempLobe_Rt	78.4% ± 4.0%	**19.8 ± 5.6**	**2.0 ± 0.9**	**79.2% ± 3.1%**	20.1 ± 6.4	2.1 ± 0.8
Thyroid_Lt	72.8% ± 10.3%	12.8 ± 6.2	1.9 ± 1.3	**74.2% ± 10.6%**	**12.2 ± 6.9**	**1.8 ± 1.4**
Thyroid_Rt	73.7% ± 10.9%	10.0 ± 4.4	1.6 ± 1.1	**75.9% ± 10.0%**	**9.4 ± 4.8**	**1.5 ± 1.1**
TMJ_Lt	68.9% ± 15.6%	10.0 ± 6.5	2.3 ± 1.6	**73.2% ± 11.9%**	**5.1 ± 2.1**	**0.8 ± 0.5**
TMJ_Rt	68.7% ± 15.5%	10.0 ± 7.3	2.5 ± 1.9	**72.4% ± 12.3%**	**5.2 ± 2.1**	**0.9 ± 0.6**
**Average**	72.4%	9.5	1.8	**76.9%**	**8.1**	**1.3**

Note: Bold and highlighted values represent the best performance and statistically significant improvements calculated using Wilcoxon matched-pairs signed rank test as compared between UaNet and SOARS, respectively. Statistical significance is set at two-tailed $p<0.05$. When the mandible is considered as a single OAR instead of left and right mandible, SOARS achieves the mean DSC, HD and ASD of 89.0%, 7.7mm, and 1.2mm, respectively. Similarly, when the thyroid is considered as a single OAR instead of left and right thyroid, SOARS achieves the mean DSC, HD and ASD of 75.0%, 10.8mm, and 1.7mm, respectively.

Supplementary Table 7. Dice similarity coefficient comparison with the previous published results on MICCAI2015 testing dataset. SOARS_Retrain achieves the top performance with the best performance in 8 out of 9 OARs (in bold). SOARS_Inference represents the results of directly inferencing on the MICCAI 2015 testing cases using the CGMH trained SOARS model. SOARS_Retrain refers to retrain SOARS using the MICCAI 2015 training cases, then, apply the retrained model to the MICCAI 2015 testing cases.

	Brainstem	Mandible	Optic Chiasm	Optic Nerve		Parotid		SMG		AVG.
				left	right	left	right	left	right	
Ren et al., 2018	-	-	58.0 ± 17.0	72.0 ± 8.0	70.0 ± 9.0	-	-	-	-	-
Wang, et al., 2018	**90.0 ± 4.0**	94.0 ± 1.0	-	-	-	83.0 ± 6.0	83.0 ± 6.0	-	-	-
Nikolov et al.[20]	79.5 ± 7.8	94.0 ± 2.0	-	71.6 ± 5.8	69.7 ± 7.1	86.7 ± 2.8	85.3 ± 6.2	76.0 ± 8.9	77.9 ± 7.4	-
Tong et al.[19]	87.0 ± 3.0	93.7 ± 1.2	58.4 ± 10.3	65.3 ± 5.8	68.9 ± 4.7	83.5 ± 2.3	83.2 ± 1.4	75.5 ± 6.5	81.3 ± 6.5	77.4
Harrison et al.[28]	87.2 ± 2.5	93.1 ± 1.8	55.6 ± 14.1	72.6 ± 4.6	71.2 ± 4.4	87.7 ± 1.8	87.8 ± 2.3	80.6 ± 5.5	80.7 ± 6.1	79.6
AnatomyNet [21]	86.7 ± 2.0	92.5 ± 2.0	53.2 ± 15.0	72.1 ± 6.0	70.6 ± 10	88.1 ± 2.0	87.3 ± 4.0	81.4 ± 4.0	81.3 ± 4.0	79.2
FocusNet [23]	87.5 ± 2.6	93.5 ± 1.9	59.6 ± 18.1	73.5 ± 9.6	74.4 ± 7.2	86.3 ± 3.6	87.9 ± 3.1	79.8 ± 8.1	80.1 ± 6.1	80.3
UaNet [24]	87.5 ± 2.5	95.0 ± 0.8	61.5 ± 10.2	74.8 ± 7.1	72.3 ± 5.9	88.7 ± 1.9	87.5 ± 5.0	82.3 ± 5.2	81.5 ± 4.5	81.2
SOARS_Inference	87.7 ± 2.5	94.8 ± 1.6	61.8 ± 13.1	72.5 ± 8.1	72.1 ± 9.5	88.1 ± 2.5	87.7 ± 3.2	79.7 ± 7.5	79.1 ± 7.9	80.4
SOARS_Retrain	88.6 ± 2.7	**96.6 ± 0.8**	**69.2 ± 9.8**	**75.8 ± 6.1**	**75.2 ± 4.8**	**88.9 ± 2.2**	**88.6 ± 4.8**	**84.5 ± 6.9**	**85.1 ± 5.8**	**83.6**

Note: Bold values represent the best quantitative performance as compared between different methods.

Supplementary Table 8. Quantitative results on the StructSeg 2019 dataset using 5-fold cross validation evaluation. The proposed SOARS outperforms the previous leading approaches UaNet and nnUNet in nearly all metrics across different OARs. DSC and HD represent Dice similarity coefficient and Hausdorff distance, respectively.

OARs	UaNet		nnUNet		SOARS	
	DSC	HD (mm)	DSC	HD (mm)	DSC	HD (mm)
BrainStem	85.3% ± 4.7%	6.6 ± 2.1	87.1% ± 4.1%	6.1 ± 2.3	**87.7% ± 3.6%**	**5.7 ± 2.1**
Eye_Lt	88.2% ± 5.2%	3.7 ± 1.3	**89.5% ± 2.7%**	3.7 ± 0.8	89.2% ± 2.8%	**3.4 ± 0.6**
Eye_Rt	88.3% ± 3.6%	3.9 ± 1.2	**89.1% ± 2.5%**	3.6 ± 1.0	88.9% ± 2.7%	**3.5 ± 0.9**
InnerEar_Lt	83.4% ± 4.2%	**4.1 ± 1.5**	82.8% ± 4.0%	4.3 ± 2.3	**86.4% ± 3.7%**	4.2 ± 1.4
InnerEar_Rt	83.4% ± 6.4%	**4.0 ± 1.3**	83.0% ± 5.2%	4.5 ± 2.9	**86.5% ± 4.8%**	4.1 ± 1.1
Lens_Lt	72.1% ± 10.1%	**2.8 ± 1.3**	74.5% ± 7.9%	2.8 ± 1.1	**75.8% ± 8.6%**	2.8 ± 0.7
Lens_Rt	71.0% ± 11.6%	2.9 ± 1.0	72.7% ± 10.1%	**2.6 ± 0.8**	**75.4% ± 8.7%**	2.9 ± 0.8
Mandible_Lt	91.0% ± 2.2%	8.7 ± 4.5	91.0% ± 2.0%	8.2 ± 2.4	91.0% ± 2.0%	**6.8 ± 2.3**
Mandible_Rt	90.7% ± 3.1%	9.1 ± 7.3	91.1% ± 2.1%	9.2 ± 10.8	**91.2% ± 2.0%**	**7.6 ± 3.0**
MidEar_Lt	79.1% ± 9.1%	9.3 ± 4.7	80.1% ± 9.5%	8.7 ± 4.2	**81.5% ± 5.8%**	**7.8 ± 4.5**
MidEar_Rt	78.2% ± 9.1%	8.1 ± 4.4	80.0% ± 8.8%	7.1 ± 3.0	**81.6% ± 6.2%**	**6.6 ± 3.6**
OpticChiasm	55.9% ± 12.1%	6.9 ± 2.5	53.5% ± 13.1%	6.9 ± 2.4	**61.2% ± 11.2%**	**4.8 ± 1.8**
OpticNerve_Lt	67.0% ± 9.3%	4.6 ± 1.7	67.6% ± 8.8%	4.6 ± 1.6	**71.7% ± 10.4%**	**2.7 ± 1.0**
OpticNerve_Rt	66.4% ± 11.1%	5.0 ± 2.4	66.8% ± 10.3%	4.6 ± 2.0	**70.4% ± 8.7%**	**2.6 ± 0.9**
Parotid_Lt	84.2% ± 5.9%	10.9 ± 4.9	85.6% ± 3.9%	11.3 ± 4.7	**85.8% ± 3.5%**	**9.8 ± 3.7**
Parotid_Rt	83.9% ± 5.6%	12.2 ± 6.4	85.7% ± 3.5%	12.2 ± 6.2	**85.8% ± 3.4%**	**11.2 ± 5.8**
Pituitary	62.0% ± 14.2%	3.7 ± 0.9	59.9% ± 16.9%	4.1 ± 1.3	**64.2% ± 15.4%**	**3.6 ± 1.2**
SpinalCord	81.3% ± 7.9%	4.6 ± 1.7	82.6% ± 3.4%	4.4 ± 1.9	**83.2% ± 3.2%**	**4.4 ± 2.0**
TempLobe_Lt	85.1% ± 5.5%	12.0 ± 4.8	86.6% ± 4.7%	12.8 ± 5.2	**86.8% ± 4.7%**	**11.1 ± 3.7**
TempLobe_Rt	85.2% ± 5.5%	**11.8 ± 4.8**	85.8% ± 4.5%	13.7 ± 4.0	**86.1% ± 4.4%**	12.9 ± 6.1
TMJ_Lt	72.6% ± 10.4%	5.6 ± 2.6	74.0 ± 10.4%	5.7 ± 2.9	**74.7 ± 6.6%**	**5.0 ± 1.3**
TMJ_Rt	73.9% ± 9.0%	5.4 ± 2.5	73.0 ± 8.5%	5.4 ± 2.7	**75.6 ± 5.8%**	**4.6 ± 1.3**
Average	78.6%	6.6	79.2%	6.7	**80.9%**	**5.8**

Note: Bold values represent the best quantitative performance as compared between UaNet, nnUNet and SOARS, respectively.

Supplementary Table 9. Quantitative clinical dosimetric accuracy ($\mathrm{Diff}^{\mathrm{clinical}}_{\mathrm{mean\ dose}}$ and $\mathrm{Diff}^{\mathrm{clinical}}_{\mathrm{max\ dose}}$) comparison between SOARS, SOARS-revised and human reader contours on 10 randomly chosen patients from the multi-user testing dataset. Dose errors are calculated by generating new IMRT plans based on each OAR contouring permutation, then overlying the gold standard OAR contours on top of each plan. Differences in mean dose and differences in maximum dose are calculated using the Equation (8) or (9) of revised version, respectively.

Clinical dosimetric accuracy ($\mathrm{Diff}^{\mathrm{clinical}}_{\mathrm{mean\ dose}}$ and $\mathrm{Diff}^{\mathrm{clinical}}_{\mathrm{max\ dose}}$)						
OARs	human reader		SOARS		SOARS-revised	
	mean dose diff	max dose diff	mean dose diff	max dose diff	mean dose diff	max dose diff
BrainStem	1.9% ± 1.2%	4.7% ± 7.1%	**1.5% ± 0.6%**	5.5% ± 4.8%	**1.3% ± 1.0%**	5.4% ± 5.3%
Eye_Lt	2.3% ± 1.7%	4.2% ± 2.6%	3.4% ± 2.2%	5.3% ± 3.7%	3.6% ± 2.5%	4.7% ± 2.8%
Eye_Rt	4.0% ± 2.8%	3.1% ± 3.5%	**3.3% ± 3.7%**	**2.3% ± 2.4%**	**3.8% ± 3.9%**	3.2% ± 2.6%
Lens_Lt	2.3% ± 2.4%	5.8% ± 6.9%	2.3% ± 2.0%	**4.9% ± 5.5%**	2.5% ± 2.0%	5.8% ± 5.4%
Lens_Rt	4.6% ± 4.9%	7.3% ± 7.3%	**4.5% ± 4.3%**	**4.6% ± 6.7%**	**4.4% ± 3.9%**	**4.7% ± 6.1%**
OpticChiasm	7.5% ± 9.3%	5.9% ± 8.1%	**5.0% ± 5.1%**	**2.9% ± 3.5%**	**4.5% ± 4.4%**	**1.9% ± 1.4%**
OpticNerve_Lt	13.9% ± 9.8%	5.9% ± 6.9%	**11.7% ± 6.3%**	**2.7% ± 4.1%**	**11.3% ± 6.7%**	**2.7% ± 3.3%**
OpticNerve_Rt	11.7% ± 9.1%	4.6% ± 5.5%	13.5% ± 10.1%	**2.3% ± 2.0%**	14.6% ± 12.2%	4.2% ± 3.4%
Parotid_Lt	5.2% ± 3.3%	3.2% ± 2.6%	**4.8% ± 3.3%**	**2.5% ± 2.2%**	**4.9% ± 3.4%**	**2.7% ± 3.0%**
Parotid_Rt	4.1% ± 3.6%	1.6% ± 1.0%	**4.0% ± 2.9%**	**1.2% ± 0.6%**	**3.9% ± 3.1%**	**1.4% ± 1.1%**
SpinalCord	6.9% ± 4.2%	6.0% ± 9.1%	**5.8% ± 3.2%**	**4.7% ± 7.4%**	**5.4% ± 3.1%**	**3.4% ± 6.4%**
TMJ_Lt	2.8% ± 2.9%	2.1% ± 1.7%	2.8% ± 2.7%	**2.0% ± 2.1%**	**1.8% ± 1.6%**	2.4% ± 2.2%
TMJ_Rt	2.0% ± 2.2%	1.7% ± 1.7%	2.4% ± 2.3%	2.5% ± 1.9%	2.9% ± 2.0%	2.6% ± 2.2%
**Average**	5.3%	4.1%	**5.0%**	**3.4%**	**5.0%**	**3.5%**

Note: mean dose diff and max dose diff represent the difference in mean dose and difference in maximum dose, respectively. SOARS and SOARS-revised results are compared to human reader results, and bold values represent quantitatively better performance.

Supplementary Table 10. Results of the human observer variation for evaluating the OAR editing efforts in the blind user study using 30 multi-user patients from FAH-ZU.

	Number of OAR required editing (%)	Number of OAR without editing (%)	Total number of OAR contours assessed
Gold-standard contour	20 (15%)	110 (85%)	130
SOAR contour	54 (43%)	71 (57%)	125
Human reader's contour	74 (55%)	61 (45%)	135

Supplementary References

1 Isensee, F., Jaeger, P. F., Kohl, S. A., Petersen, J. & Maier-Hein, K. H. nnU-Net: a self-configuring method for deep learning-based biomedical image segmentation. *Nature methods* **18**, 203-211 (2021).

2 Ulyanov, D., Vedaldi, A. & Lempitsky, V. Instance normalization: The missing ingredient for fast stylization. *arXiv preprint arXiv:1607.08022* (2016).

3 Liu, H., Simonyan, K. & Yang, Y. Darts: Differentiable architecture search. *arXiv preprint arXiv:1806.09055* (2018).

4 Liu, C. *et al.* Auto-deeplab: Hierarchical neural architecture search for semantic image segmentation. *Proceedings of the IEEE/CVF Conference on Computer Vision and Pattern Recognition.* 82-92 (2019).
