# Supplementary material for: Comprehensive and clinically accurate head and neck cancer organs-at-risk delineation on a multi-institutional study
Source: Nat Commun. 2022 Oct 17;13:6137. doi: 10.1038/s41467-022-33178-z (PMC9576793; doi:10.1038/s41467-022-33178-z)
Supplement: Supplementary file 2 — Reporting Summary [file 41467_2022_33178_MOESM2_ESM.pdf]

nature portfolio

Corresponding author(s): Tsung-Ying Ho

Last updated by author(s): Sep 7, 2022

Reporting Summary

Nature Portfolio wishes to improve the reproducibility of the work that we publish. This form provides structure for consistency and transparency in reporting. For further information on Nature Portfolio policies, see our Editorial Policies and the Editorial Policy Checklist.

Statistics

For all statistical analyses, confirm that the following items are present in the figure legend, table legend, main text, or Methods section.

n/a	Confirmed	
☐	☒	The exact sample size (n) for each experimental group/condition, given as a discrete number and unit of measurement
☐	☒	A statement on whether measurements were taken from distinct samples or whether the same sample was measured repeatedly
☐	☒	The statistical test(s) used AND whether they are one- or two-sided<br>*Only common tests should be described solely by name; describe more complex techniques in the Methods section.*
☒	☐	A description of all covariates tested
☒	☐	A description of any assumptions or corrections, such as tests of normality and adjustment for multiple comparisons
☐	☒	A full description of the statistical parameters including central tendency (e.g. means) or other basic estimates (e.g. regression coefficient) AND variation (e.g. standard deviation) or associated estimates of uncertainty (e.g. confidence intervals)
☐	☒	For null hypothesis testing, the test statistic (e.g. F, t, r) with confidence intervals, effect sizes, degrees of freedom and P value noted<br>*Give P values as exact values whenever suitable.*
☒	☐	For Bayesian analysis, information on the choice of priors and Markov chain Monte Carlo settings
☒	☐	For hierarchical and complex designs, identification of the appropriate level for tests and full reporting of outcomes
☒	☐	Estimates of effect sizes (e.g. Cohen's d, Pearson's r), indicating how they were calculated

Our web collection on statistics for biologists contains articles on many of the points above.

Software and code

Policy information about availability of computer code

Data collection	No software was used for data collection.
Data analysis	The code for analysis in this study was written using Python3.6, the deep learning models were implemented using Pytorch v1.4.<br>The quantitative results were analyzed using R 4.1.0. The baseline UNet used in this study is implemented in the nnUNet deep learning framework, available at https://github.com/MIC-DKFZ/nnUNet. The codes used for inference and result performance evaluation is available at: https://doi.org/10.5281/zenodo.6998392.

For manuscripts utilizing custom algorithms or software that are central to the research but not yet described in published literature, software must be made available to editors and reviewers. We strongly encourage code deposition in a community repository (e.g. GitHub). See the Nature Portfolio guidelines for submitting code & software for further information.

Data

Policy information about availability of data

All manuscripts must include a data availability statement. This statement should provide the following information, where applicable:

- Accession codes, unique identifiers, or web links for publicly available datasets
- A description of any restrictions on data availability
- For clinical datasets or third party data, please ensure that the statement adheres to our policy

The imaging data from internal and external institutions are not publicly available due to the data privacy and restricted permissions of the current study. Researchers who are interested in our work can request access from the corresponding author upon academic purposes. Sample testing imaging data from two public HN OAR datasets can be directly downloaded from https://www.imagenglab.com/newsite/pddca and https://structseg2019.grand-challenge.org.

Field-specific reporting

Please select the one below that is the best fit for your research. If you are not sure, read the appropriate sections before making your selection.

- [x] Life sciences
- [ ] Behavioural & social sciences
- [ ] Ecological, evolutionary & environmental sciences

For a reference copy of the document with all sections, see nature.com/documents/nr-reporting-summary-flat.pdf

Life sciences study design

All studies must disclose on these points even when the disclosure is negative.

Sample size	The sample size is determined by collecting head & neck cancer patients who received RT as their primary treatment from Chang Gung Memorial Hospital (2014-2020), First Affiliated Hospital of Xi'an Jiaotong University (2017-2019), and First Affiliated Hospital of Zhejiang University (2014-2020), Gansu Provincial Hospital (2018-2020), Huadong Hospital Affiliated of Fudan University (2017-2020), Southern Medical University (2017-2020).
Data exclusions	Having surgery, missing image data file, poor image quality such as severe metal artifacts.
Replication	We confirm that our attempts for replication were all successful.
Randomization	The multi-user testing dataset is formed by randomly selecting 30 nasopharyngeal cancer patients from the First Affiliated Hospital of Zhejiang University and 20 nasopharyngeal cancer patients from Southern Medical University.
Blinding	There were no blinding in this study. This study is an image-based algorithm validation study. No more blind study design was needed.

Reporting for specific materials, systems and methods

We require information from authors about some types of materials, experimental systems and methods used in many studies. Here, indicate whether each material, system or method listed is relevant to your study. If you are not sure if a list item applies to your research, read the appropriate section before selecting a response.

Materials & experimental systems

n/a	Involved in the study	
x		Antibodies
x		Eukaryotic cell lines
x		Palaeontology and archaeology
x		Animals and other organisms
	x	Human research participants
x		Clinical data
x		Dual use research of concern

Methods

n/a	Involved in the study	
x		ChIP-seq
x		Flow cytometry
x		MRI-based neuroimaging

Human research participants

Policy information about studies involving human research participants

Population characteristics	This multi-institutional retrospective study contained, in total, 1503 head and neck cancer patients. The training dataset contained 176 patients (160 male) whose median and interquartile age were 54 [48-61]. The internal testing dataset contained 326 patients (284 male) whose median and interquartile age were 54 [49-62]. The external testing dataset contained 1001 patients (725 male), whose median and interquartile age were 57 [49-66].
Recruitment	There was no patient recruitment involved since this is a retrospective study. The patients' image data were collected from six institutions: Chang Gung Memorial Hospital, First Affiliated Hospital of Xi'an Jiaotong University, First Affiliated Hospital of Zhejiang University, Gansu Provincial Hospital, Huadong Hospital Affiliated of Fudan University, and Southern Medical University, which contain de-identified patient information from each institution.
Ethics oversight	The multi-center patients recruitment in this study were under each institutional review board approval, including Chang Gung Memorial Hospital, First Affiliated Hospital of Xi'an Jiaotong University, First Affiliated Hospital of Zhejiang University, Gansu Provincial Hospital, Huadong Hospital Affiliated of Fudan University, and Southern Medical University. Requirements to obtain informed consent were waived.

Note that full information on the approval of the study protocol must also be provided in the manuscript.
